# Assessment of tissue perfusion of pancreatic cancer as potential imaging biomarker by means of Intravoxel incoherent motion MRI and CT perfusion: correlation with histological microvessel density as ground truth

**DOI:** 10.1186/s40644-021-00382-x

**Published:** 2021-01-19

**Authors:** Philipp Mayer, Franziska Fritz, Marco Koell, Stephan Skornitzke, Frank Bergmann, Matthias M. Gaida, Thilo Hackert, Klaus Maier-Hein, Frederik B. Laun, Hans-Ulrich Kauczor, Lars Grenacher, Miriam Klauß, Wolfram Stiller

**Affiliations:** 1 Clinic for Diagnostic and Interventional Radiology (DIR), Heidelberg University Hospital, Im Neuenheimer Feld 420, 69120 Heidelberg, Germany; 2 Institute of Pathology, Heidelberg University Hospital, Im Neuenheimer Feld 224, 69120 Heidelberg, Germany; 3 Institute of Pathology, University Medical Center Mainz, Johannes Gutenberg-University Mainz, Langenbeckstrasse 1, 55131 Mainz, Germany; 4 Department of General, Visceral, and Transplantation Surgery, Heidelberg University Hospital, Im Neuenheimer Feld 420, 69120 Heidelberg, Germany; 5 Department of Medical Imaging Computing, German Cancer Research Center, Im Neuenheimer Feld 223, 69120 Heidelberg, Germany; 6 Department of Medical Physics in Radiology, German Cancer Research Center, Im Neuenheimer Feld 223, 69120 Heidelberg, Germany; 7 Institute of Radiology, University Hospital Erlangen, Maximiliansplatz 3, 91054 Erlangen, Germany; 8 Conradia Radiology Munich, Augustenstraße 115, 80798 Munich, Germany

**Keywords:** Pancreatic ductal adenocarcinoma, X-ray computed tomography, Diffusion magnetic resonance imaging, Microvessels

## Abstract

**Background/objectives:**

The aim of this study was to compare intravoxel incoherent motion (IVIM) diffusion weighted (DW) MRI and CT perfusion to assess tumor perfusion of pancreatic ductal adenocarcinoma (PDAC).

**Methods:**

In this prospective study, DW-MRI and CT perfusion were conducted in nineteen patients with PDAC on the day before surgery. IVIM analysis of DW-MRI was performed and the parameters perfusion fraction *f*, pseudodiffusion coefficient D*, and diffusion coefficient D were extracted for tumors, upstream, and downstream parenchyma. With a deconvolution-based analysis, the CT perfusion parameters blood flow (BF) and blood volume (BV) were estimated for tumors, upstream, and downstream parenchyma. In ten patients, intratumoral microvessel density (MVD_tumor_) and microvessel area (MVA_tumor_) were analyzed microscopically in resection specimens. Correlation coefficients between IVIM parameters, CT perfusion parameters, and histological microvessel parameters in tumors were calculated. Receiver operating characteristic (ROC) analysis was performed for differentiation of tumors and upstream parenchyma.

**Results:**

*f*_tumor_ significantly positively correlated with BF_tumor_ (r = 0.668, *p* = 0.002) and BV_tumor_ (r = 0.672, p = 0.002). There were significant positive correlations between *f*_tumor_ and MVD_tumor_/ MVA_tumor_ (r ≥ 0.770, *p* ≤ 0.009) as well as between BF_tumor_ and MVD_tumor_/ MVA_tumor_ (r ≥ 0.697, *p* ≤ 0.025). Correlation coefficients between *f*_tumor_ and MVD_tumor_/ MVA_tumor_ were not significantly different from correlation coefficients between BF_tumor_ and MVD_tumor_/ MVA_tumor_ (*p* ≥ 0.400). Moreover, *f*, BF, BV, and permeability values (PEM) showed excellent performance in distinguishing tumors from upstream parenchyma (area under the ROC curve ≥0.874).

**Conclusions:**

The study shows that IVIM derived *f*_tumor_ and CT perfusion derived BF_tumor_ similarly reflect vascularity of PDAC and seem to be comparably applicable for the evaluation of tumor perfusion for tumor characterization and as potential quantitative imaging biomarker.

**Trial registration:**

DRKS, DRKS00022227, Registered 26 June 2020, retrospectively registered. https://www.drks.de/drks_web/navigate.do?navigationId=trial. HTML&TRIAL_ID=DRKS00022227.

**Supplementary Information:**

The online version contains supplementary material available at 10.1186/s40644-021-00382-x.

## Introduction

Pancreatic ductal adenocarcinoma (PDAC) is notorious for its exceptionally high mortality with an estimated global mortality rate of 98% [1]. It has been reported that the complex tumor biology with the distinct microenvironment of the cancer has important influence on the prognosis of PDAC [2–4]. One of the factors which are known to be associated with prognosis in malignomas is tumor vascularity quantified histologically by the intratumor microvessel density (i.e. number of microvessels per tumor area, MVD). In various studies on PDAC, high MVD was associated with higher risk for liver and lymph node metastases as well as short survival time [5–12].

Tissue perfusion is regarded as a non-invasive marker for tumor vascularity and has been evaluated as biomarker of drug action in early phase trials for the treatment of various solid tumors [13], including PDAC [14]. Moreover, tissue perfusion was reported useful for the differentiation of local recurrence from unspecific postoperative alterations after PDAC resection [15].

For measurement of tumor tissue perfusion, the intravoxel incoherent motion (IVIM) model for diffusion weighted (DW) magnetic resonance imaging (MRI) as well as computed tomography (CT) perfusion have gained increasing interest during the past years. The IVIM model enables to extract parameters of both pure extravascular molecular diffusion and perfusion from DW MRI data [16]. CT perfusion measures tissue perfusion through the analysis of temporal changes in attenuation during the first pass of a bolus of iodinated contrast material [13].

Many previous studies have reported direct correlations between CT perfusion- derived parameters and MVD in other solid tumors [17], including pancreatic neuroendocrine neoplasms (PNEN) [18]. However, studies which correlate the histologically derived MVD with IVIM-derived parameters, or correlate IVIM-derived parameters and CT perfusion- derived parameters are still quite rare. In a recent study, Klauß et al. showed a good correlation between the IVIM-derived perfusion fraction *f* and the MVD in PDAC and PNEN [19]. A previous study failed to show a correlation between IVIM-derived parameters and CT perfusion-derived parameters in hepatocellular carcinomas [20].

This raises the question if CT perfusion and IVIM MRI are comparably applicable for the assessment of tissue perfusion in solid tumors, or whether one method should be preferred over the other.

Therefore, the aim of our study was to compare IVIM DW MRI and CT perfusion to assess tumor perfusion of PDAC.

## Methods

### Study design, patient population and demographics

The study protocol of our prospective study was approved by our institutional review board and informed consent was obtained from the patients before the examination. Between August 2014 and July 2015, 23 patients (13 females and 10 males; mean age, 63.2 ± 7.9 years [range: 50–79 years]) with detection of a mass suspicious of PDAC in a previous CT and/or MRI and scheduled surgery with potentially curative intent were consecutively enrolled in our study. In all patients, indication for surgery had been made prior to and independently from the present study. The patients were scanned and evaluated prospectively, first with IVIM DW MRI, and second with CT perfusion, on the day before surgery.

Exclusion criteria were: general contraindications for the application of iodinated contrast agents, MR-unsafe foreign bodies, previous treatment for pancreatic carcinoma, inability to reproduce the breathing technique (see below), and/or denial of consent.

There was no histologically confirmed diagnosis at the time of inclusion in our study.

Out of the 23 total patients, the final histopathological diagnosis was PDAC in 20 patients, PNEN in one patient, anaplastic carcinoma in one patient, and mass-forming chronic pancreatitis in one patient. The three patients with histopathological diagnosis other than PDAC were excluded from our study. In one patient with PDAC the tumor area was missed in CT perfusion, therefore this patient was also excluded. CT perfusion and MRI diffusion could be evaluated in all remaining 19 patients. In 16 of these 19 patients, the tumor was located in the pancreatic head while 3 patients had a tumor in the pancreatic body and/ or tail. Among these 19 patients, resection was not possible and/or indicated due to infiltration of the superior mesenteric artery (SMA) in 1 patient, due to peritoneal metastases in 2 patients, and due to hepatic metastases in 1 patient. In these 4 patients in whom tumor resection was not performed, histological diagnosis was established from intraoperative biopsies from the part where the tumor infiltrated the SMA, or from the peritoneal/ hepatic metastases, respectively. In all 15 other patients, histological diagnosis was established from the resected primary tumor.

### Null hypothesis and alternative hypothesis

The null hypothesis of the study is that the correlation of the perfusion fraction *f* from IVIM DW MRI with MVD/ microvessel area (MVA) is not different from the correlation of blood flow (BF) from CT perfusion with MVD/ MVA. The alternative hypothesis of the study is that the correlation of *f* with MVD/ MVA is different from the correlation of BF with MVD/ MVA.

### MR imaging and Post-processing

MR imaging was performed using a 1.5 T scanner (Magnetom Avanto, Siemens Medical Solutions) with a 6-element body-phased array coil and a 24-channel spine array coil. The pancreatic MR imaging protocol consisted of anatomic imaging sequences and diffusion weighted imaging (DWI) with 9 b-values (0, 50, 100, 150, 200, 300, 400, 600, and 800 s/mm^2^, summarized in Table 1). This combination of b-values was chosen since it had proven feasible for assessment of tissue perfusion in correlation to MVD in PDAC [19]. b-Values larger than 800 s/mm^2^ were not chosen to minimize the kurtosis effect that becomes increasingly important at larger b-values [21].

**Table 1:** Parameters of MR imaging

Sequence type	Breathing position	Coverage	Orientation	TR [ms]	TE [ms]	Acquisition matrix	Slice thickness/ gap [mm]	Pixel bandwidth [Hz]	
**1) Anatomic MR imaging (performed in every patient)**									
T1-weighted in/opposed phase	Inspiratory breath-hold	Upper abdomen	Transverse	115	2.27 and 4.78	320 × 272	5 / 1	445	
HASTE-IR T2-weighted	Inspiratory breath-hold	Upper abdomen	Coronal	1000	80	256 × 230	6 / 0.6	545	
HASTE T2-weighted	Expiratory breath-hold	Upper abdomen	Transverse	680	95	320 × 320	4 / 0.4	505	
**2) Diffusion weighted MR imaging (performed in every patient)**									
ss-EPI	Expiratory breath-hold	Pancreas	Transverse	2200	58	130 × 92	5 / 0.25	2260	**Pixel spacing:** 2.7 mm/ 2.7 mm; **Number of acquired slices per b-value**: 14; **b-values [s/mm**^**2**^**]:** 0, 50, 100, 150, 200, 300, 400, 600, and 800; **Number of excitations:** 1 for b = 0 s/mm^2^, 2 for every other b-value; **Number of** ***diffusion*-encoding** ***gradient directions:*** *3;* **K-space based parallel imaging technique (GRAPPA); acceleration factor:** 2; **Fat saturation technique:** spectral fat saturation.
The acquisition was separated into blocks (*b*_0_, *b*_50_), (*b*_0_, *b*_100_) … (*b*_0_, *b*_800_). Each block was acquired in a single breath-hold in expiration (TA = 22 s) to avoid motion artifacts. No registration for correction of patient breathing-motion was applied.									

The pancreatic MR imaging protocol consisted of 1) anatomic imaging sequences, and 2) diffusion weighted imaging with 9 b-values. An experienced radiologist directly involved in the study was always present during MR imaging. Abbreviations: n.a.: not applicable, fs: fat saturation, HASTE: Half-Fourier-Acquired Single-shot Turbo spin Echo, ss-EPI: Single-shot Echo-Planar Imaging, TE: echo time, TR: repetition time

MITK Diffusion software version 2017.07 (Medical Imaging Interaction Toolkit, DKFZ Heidelberg, www.MITK.org) was used for post-processing of DWI data [22]. Among several possible approaches described by Klaasen et al. [23], the following approach according to the IVIM model was chosen to calculate the perfusion fraction *f*, pseudodiffusion coefficient D*, and diffusion coefficient D, as previously described [19] (corresponding to Klaasen’s model no. 3). The signal was averaged within a region of interest for each b-value. Then the equation

$$ \frac{S_b}{S_0}=\left(1-f\right)\ast \exp \left(-b\ast D\right)+f\ast \exp \left(-b\ast \left(D+D\ast \right)\right) $$

was fitted to the data. Here, S_b_ stands for the signal with diffusion weighting and S_0_ for the signal without diffusion weighting. Measurements at b-values greater than 170 s/mm^2^ were used in a first step to estimate f and D. D* was then calculated in a second step by using exhaustive search.

Quantitative analysis of DWI was performed independently by two radiologists with at least 5 years of experience in abdominal imaging each (P.M. and F.F.), blinded to the other radiologist’s analysis and other patient information. Free-hand volumes of interest (VOIs) were drawn encompassing the tumor on DW images. The exact anatomical outline of the tumor was determined with the help of conventional CT images and/ or conventional biliary-pancreatic MR images. Calcifications (as detected by CT) and cystic/ necrotic tumor areas without enhancement (as detected by contrast enhanced CT) were excluded from the VOIs. When possible, upstream and downstream pancreatic parenchyma also was segmented by Reader 1 (P.M.). The reported values of *f*, D, and D* were derived from the generated VOIs in all cases.

### CT imaging and Post-processing

Immediately after MR imaging, the patients were examined with CT imaging.

All examinations were carried out with a 2 × 64-slice CT scanner (Somatom Definition Flash; Siemens Medical Solutions), using the hydro-CT-technique [24]. Patients were placed on the CT table in an oblique, 30°, right-sided down position. The acquisition protocol is summarized in Table 2.

**Table 2:** Parameters of CT imaging

Phase	Breathing position	Anatomical coverage	Tube voltage [kV_**p**_]	Reference tube current (eff.) [mAs]	Delay [s]	Rotation time [s]	Number of Acquisitions	Collimation [mm]	Slice thickness (recon.) [mm]	Pixel spacing [mm]	Reconstruction Kernel
**1) Standard 3-phasic CT scan (only performed if patient didn’t have an in-house CT scan of the abdomen within previous 4 weeks)** Application of 80 ml of nonionic iodinated contrast agent; chaser bolus: 40 ml saline solution; bolus tracking in suprarenal aorta (threshold: 100 HU).											
**Native**	Inspiratory breath-hold	Upper abdomen	120	210	4	0.5	Helical	2 × 64 × 0.6	3	Variable	I30f
**Arterial**	Inspiratory breath-hold	Upper abdomen	120	210	10	0.5	Helical	2 × 64 × 0.6	3	Variable	I30f
**Portal-venous**	Inspiratory breath-hold	Whole abdomen	120	210	50	0.5	Helical	2 × 64 × 0.6	3	Variable	I30f
15 min break for contrast medium clearance (as applied in previous studies [35, 42]). Patient remains on CT table. Patient is instructed to a shallow breathing technique for reducing motion artefacts in the perfusion acquisition.											
**2) Native scan for verification of the correct position of the examination volume for CT perfusion imaging (performed in every patient)**											
**Native**	Shallow breathing	Pancreas	120	210	n.a.	0.5	Helical	2 × 64 × 0.6	3	Variable	I30f
**3) CT perfusion imaging (performed in every patient)** Application of 80 ml of nonionic iodinated contrast agent; chaser bolus: 40 ml saline solution; flow rate: 5 ml/s.											
CT perfusion imaging *	Shallow breathing	Tumor area	80	270	13	0.5 (full rotation); 1.5 (cycle time)	34	32 × 0.6	3 × 5.0	0.6 / 0.6	B30f

The pancreatic CT imaging protocol consisted of 1) a standard 3-phasic CT acquisition which was only performed if the patient didn’t have an in-house CT examination of the abdomen within previous 4 weeks, 2) a native acquisition for verification of the correct position of the examination volume for CT perfusion imaging (performed in every patient), and 3) CT perfusion imaging (performed in every patient). All CT acquisitions were performed with automated tube-current modulation. CT acquisitions from examination series grouped under point 1) and point 2) were performed with automated selection of tube voltage (reference tube voltage listed). An experienced radiologist directly involved in the study was always present during CT imaging. Abbreviations: n.a.: not applicable

Perfusion data were analyzed with a body-perfusion CT-tool (Body-PCT, Siemens Medical Solutions) at a multimodality workplace with the syngo.via imaging software version VB 30 (Siemens Medical Solutions).

The baseline definition for motion correction at any time step and for segmentation at time step zero was followed by the segmentation of an organ VOI and the definition of a circular region of interest (ROI) in the aorta for vascular identification. The mean tissue time-attenuation curve was derived automatically and based on these definitions and data the color-coded parameter maps were established and confirmed.

Two radiologists with at least 5 years of experience in interpreting abdominal images each (P.M. and F.F.), independently placed polygonal VOIs encompassing the tumor, blinded to the other radiologist’s analysis and other patient information. For exact CT VOI placement, the radiologists had access to the same set of conventional CT/MR images as provided for MRI VOI placement. When possible, upstream and downstream pancreatic parenchyma was also segmented by Reader 1 (P.M.). Calcifications and cystic/ necrotic tumor areas without enhancement were excluded from the VOIs. To avoid a potential bias, the time interval between IVIM DWI analysis and CT perfusion analysis was at least 3 months for each radiologist.

Using a deconvolution model the software calculated the following parameters:

$$ BF\ \left( blood\ flow\right)\ \left[\frac{ml}{100\  ml\ast \mathit{\min}}\right] $$

$$ PEM\ (permeability)\ \left[\frac{ml}{100\  ml\ast \mathit{\min}.}\right] $$

$$ BV\ \left( blood\ volume\right)\left[\frac{ml}{100\  ml}\right] $$

The dose-length-products (DLPs) were calculated from the volume CT dose index (CTDI_vol_) - values and scan lengths:

$$ DLP={CTDI}_{vol}\ast scan\ length $$

For calculation of the effective dose (D_eff_), the DLPs were multiplied with the corresponding conversion factor for abdominal CT-examinations [25]:

$$ {D}_{eff}=\frac{0.015\  mSv}{mGy\ast cm}\ast DLP $$

Histology and immunohistochemistry for the assessment of the MVD

In resection specimens, the diagnosis of PDAC was established according to the criteria recommended by the World Health Organization (WHO) and pathological staining was provided using the Union internationale contre le cancer (UICC) criteria. Histopathological grading was based on combined assessment of growth pattern, mucin content, and mitoses. When heterogeneity (i.e. variation in the degree of differentiation) was seen, the highest grade was assigned.

TNM (tumor, node, metastasis) stages according to the 8th Edition of the UICC Manual and Grading (G) of the 15 resected tumors were as follows: T1 in 1 patient, T2 in 10 patients, T3 in 4 patients, N0 in 3 patients, N1 in 4 patients, N2 in 8 patients, M0/x in 14 patients, M1 in 1 patient. The histopathological grading was G2 in 8 patients, and G3 in 7 patients. Pathological tumor size in these 15 patients ranged from 1.8 cm to 6.1 cm (mean value: 3.35 cm ± 1.19 cm).

Because of the known tumor heterogeneity [3], tissue selection for tissue-based analysis was performed as previously described. In 10 out of 15 patients who underwent resection for PDAC, representative whole tumor slides from formalin-fixed paraffin-embedded tissue were immunostained with a CD34-specific antibody (1:25, M7165, Dako), as previously described [19]. In 4 patients, tissue slides of non-neoplastic pancreatic tissue were also immunostained with the CD34-specific antibody.

To generate digital slide images, tissue slides were scanned at 20x magnification using an Aperio slide scanner (Leica Biosystems Aperio). In 10 patients, a mean coherent tumor area of 45 mm^2^ per tumor, and in 4 patients, representative non-neoplastic pancreatic tissue were then analyzed using the Aperio Microvessel Analysis software (Leica Biosystems Aperio) [26], as previously described [19]. Plausibility was confirmed by pathologists (blinded to clinical information). MVA was calculated as the proportion of the sum of all vessel areas to the total analyzed area, MVD was calculated as mean vessel count per mm^2^.

### Statistical analysis

Statistical data analysis was performed using MedCalc version 19.2.1 (MedCalc Software Ltd., Ostend, Belgium). Spearman rank correlation coefficients between IVIM-derived parameters, CT perfusion- derived parameters, and MVD/ MVA in tumors were calculated. As proposed by Campbell and Swinscow [27], Spearman correlation was interpreted as very weak (0.00–0.19), weak (0.20–0.39), moderate (0.40–0.59), strong (0.60–0.79), or very strong (0.80–1.00). For comparing correlation coefficients, we used the test recommended by Meng et al., Steiger’s Z [28]. For a two-tailed test, Z-scores greater than 1.96 or smaller than − 1.96 are considered statistically significant. Regression analysis was applied between *f* and BF, between *f* and MVD, as well as between BF and MVD, using linear regression models. Mann-Whitney U test was used for comparison of independent continuous variables while Wilcoxon test was used for comparison of dependent continuous variables. Inter-reader reliability was assessed by using the Intra-class Correlation Coefficient (ICC) with 95% confidence intervals (CI) and applying a 2-way ICC with random raters’ assumption reproducibility. As proposed by Song et al. [29], ICC values were interpreted as poor (0.00–0.20), fair (0.21–0.40), moderate (0.41–0.60), good (0.61–0.80), or excellent (0.81–1.00). Receiver operating characteristic (ROC) curves were employed to analyze the diagnostic performance of DWI IVIM and CT perfusion parameters in distinguishing tumors from upstream parenchyma. Due to the small sample size of patients with downstream parenchyma (*n* = 5), ROC curve analysis was not performed for distinguishing tumors from downstream parenchyma. The AUCs with 95% confidence intervals (CIs) were computed. Sensitivities and specificities of the ROC curves were calculated, and the optimal cut-off values were determined. The DeLong method [30] was used for comparison of areas under the curves (AUCs). As proposed by Mandrekar [31], AUC values were interpreted as acceptable (0.70–0.79), excellent (0.80–0.89), and outstanding (0.90–1.00), while an AUC of 0.5 suggests no discriminatory ability. Significance level was set at 0.05.

## Results

### DWI IVIM parameters

Mean tumor perfusion fractions *f*_tumor_ for both radiologists ranged from 7.1 to 22.4% (median: 10.1%; interquartile range [IQR]: 8.9 to 16.6%), mean diffusion coefficients D_tumor_ ranged from 1.0 * 10^− 3^ mm^2^/s to 2.0 * 10^− 3^ mm^2^/s (median: 1.2 × 10^− 3^; IQR: 1.2 to 1.4 × 10^− 3^ mm^2^/sec), and mean pseudodiffusion coefficients D*_tumor_ ranged from 6.2 × 10^− 3^ mm^2^/s to 148.5 × 10^− 3^ mm^2^/sec (median: 17.4 × 10^− 3^; IQR: 11.4 to 131.9 × 10^− 3^ mm^2^/s).

Median DWI IVIM parameters for downstream parenchyma for reader 1 (P.M.) were: 22.5% for *f*_downstream_ (IQR: 20.2 to 25.3%), 1.7 * 10^− 3^ mm^2^/s for D_downstream_ (IQR: 1.3 to 1.7 * 10^− 3^ mm^2^/s), and 26.3 × 10^− 3^ mm^2^/s for D*_downstream_ (IQR: 13.7 to 68.1 × 10^− 3^ mm^2^/s). Median DWI IVIM parameters for upstream parenchyma for reader 1 (P.M.) were: 21.1% for *f*_upstream_ (IQR: 17.6 to 24.0%), 1.5 * 10^− 3^ mm^2^/s for D_upstream_ (IQR: 1.4 to 1.7 * 10^− 3^ mm^2^/s), and 21.9 × 10^− 3^ mm^2^/s for D*_upstream_ (IQR: 11.8 to 34.9 × 10^− 3^ mm^2^/s). DWI IVIM parameters of tumors, downstream parenchyma, and upstream parenchyma are depicted in Supplementary Figure 1 A-C).

### CT perfusion parameters and radiation exposure

Mean tumor perfusion values (BF_tumor_) for both radiologists ranged from 14.0 ml/100 ml/min to 98.9 ml/100 ml/min (median: 38.9 ml/100 ml/min; IQR: 29.0 to 66.2 ml/100 ml/min). Mean tumor blood volume values (BV_tumor_) ranged from 0.9 ml/100 ml to 11.4 ml/100 ml (median: 2.4 ml/100 ml; IQR: 1.9 to 4.6 ml/100 ml). Mean tumor permeability values (PEM_tumor_) ranged from 6.7 ml/100 ml/min to 58.0 ml/100 ml/min (median 17.8 ml/100 ml/min; IQR: 11.0 to 28.3 ml/100 ml/min).

Median CT perfusion parameters for downstream parenchyma for reader 1 (P.M.) were: 141.8 ml/100 ml/min for BF_downstream_ (IQR: 131.2 to 173.0 ml/100 ml/min), 9.9 ml/100 ml for BV_downstream_ (IQR: 8.7 to 13.3 ml/100 ml), and 60.4 ml/100 ml/min for PEM_downstream_ (IQR: 53.5 to 67.4 ml/100 ml/min). Median CT perfusion parameters for upstream parenchyma for reader 1 (P.M.) were: 117.0 for BF_upstream_ (IQR: 82.1 to 154.0 ml/100 ml/min), 8.5 ml/100 ml for BV_upstream_ (IQR: 5.7 to 14.3 ml/100 ml), and 51.4 ml/100 ml/min for PEM_upstream_ (IQR: 40.4 to 70.5 ml/100 ml/min). CT perfusion parameters of tumors, downstream parenchyma, and upstream parenchyma are depicted in Supplementary Figure 1 D-F).

Median DLPs and D_eff_ were 510.0 mGy cm/ 7.7 mSv for the standard three-phasic abdominal CT scans (*n* = 16), 116.0 mGy cm/ 1.7 mSv for the single native CT acquisitions, and 243.9 mGy cm/ 3.7 mSv for CT perfusion imaging. There were no adverse reactions to contrast media.

### Inter-reader reliability

Agreement between reader 1 (P.M.) and reader 2 (F.F.) was excellent for *f*_tumor_ (ICC = 0.8545, 95% confidence interval (CI): 0.6669 to 0.9409), BF_tumor_ (ICC = 0.8317, 95% CI: 0.6164 to 0.9315), BV_tumor_ (ICC = 0.9338, 95% CI: 0.8389 to 0.9738), PEM_tumor_ (ICC = 0.9303, 95% CI: 0.8303 to 0.9725), and good for D_tumor_ (ICC = 0.7406, 95% CI: 0.4491 to 0.8907), as well as D*_tumor_ (ICC = 0.7519, 95% CI: 0.4717 to 0.8956).

### Histopathological parameters

In the 10 patients in whom representative tumor tissue slides had been immunostained, MVD_tumor_ ranged from 21.9/mm^2^ to 103.0/mm^2^ (median: 33.2/mm^2^; IQR: 25.3 to 61.8/mm^2^, and MVA_tumor_ ranged from 0.007 to 0.045 (median: 0.014; IQR: 0.011 to 0.029).

### Comparative data analysis: DWI IVIM and CT perfusion parameters in tumors versus downstream and upstream parenchyma

*f*_upstream_ values and D_upstream_ values from Reader 1 were significantly higher than *f*_tumor_ values and D_tumor_ values (*p* ≤ 0.0010). *f*_downstream_ values were non-significantly higher than *f*_tumor_ values (*p* = 0.0625). D_downstream_ did not differ significantly from D_tumor_ (*p* = 0.6250) and D* values did not differ significantly between tumors, downstream, and upstream parenchyma (*p* ≥ 0.6250). BF, BV, and PEM values were significantly lower in tumors than in upstream parenchyma (*p* ≤ 0.0003) and non-significantly lower in tumors than in downstream parenchyma (p = 0.0625).

Receiver operating characteristic (ROC) curves (with 95% CI) of DWI IVIM and CT perfusion parameters from Reader 1 for distinguishing tumors from upstream parenchyma are presented in Supplementary Figure 2. The diagnostic accuracies of BF and BV were outstanding (AUC = 0.937; 95% CI: 0.797 to 0.991; and AUC = 0.902; 95% CI: 0.750 to 0.977). PEM and *f* showed excellent diagnostic accuracy (AUC = 0.888; 95% CI: 0.732 to 0.970; AUC = 0.874; 95% CI: 0.715 to 0.962), while D showed acceptable diagnostic accuracy (AUC = 0.737; 95% CI: 0.558 to 0.872). D* had the lowest AUC value (AUC = 0.514; 95% CI: 0.337 to 0.688). Differences in AUC values between *f* and BF/BV/PEM were not statistically significant (*p* ≥ 0.1847). Diagnostic accuracy of D* was significantly lower than diagnostic accuracy of *f*/BF/BV/PEM (*p* ≤ 0.0056). The cut-off values with the highest Youden’s indices were ≤ 10.7% for *f*, ≤ 78.2 ml/100 ml/min for BF, ≤ 3.8 ml/100 ml for BV, and ≤ 36.6 ml/100 ml/min for PEM with corresponding sensitivities of 63.2, 89.5, 68.4, and 84.2% as well as specificities of 100.0, 86.7, 100.0, and 93.3%.

### Comparative data analysis: DWI IVIM versus CT perfusion versus histological parameters

Spearman rank correlation coefficients a) between *f*_tumor_ and BF_tumor_/BV_tumor_, as well as b) between MVD_tumor_/MVA_tumor_ and *f*_tumor_/BF_tumor_/BV_tumor_ are listed in Table 3. Spearman rank correlation coefficients between all tumor DWI IVIM-parameters, CT perfusion parameters and histological microvessel parameters are summarized in *Supplementary Table* *1*.

**Table 3:** Spearman rank correlation coefficients

Spearman correlations between mean ***f***_**tumor**_ from DWI IVIM and mean CT perfusion parameters (n = 19)			
	BF_tumor_	BV_tumor_	
*f*_tumor_	0.668 *	0.672 *	
**Spearman correlations between histological microvessel parameters, mean** ***f***_**tumor**_ **from DWI IVIM and mean CT perfusion parameters (*n*** **= 10)**			
	*f*_tumor_	BF_tumor_	BV_tumor_
MVD_tumor_	0.770 *	0.697 *	0.661 *
MVA_tumor_	0.818 *	0.709 *	0.661 *

Correlation coefficients that are significantly different from zero (*p* < 0.05) are marked with *. Abbreviations: BF: blood flow, BV: blood volume, *f*: perfusion fraction, MVA: microvessel area, MVD: microvessel density

There were significant positive strong Spearman rank correlation coefficients between mean *f*_tumor_ for both readers and mean BF_tumor_/ BV_tumor_ (ρ ≥ 0.668, *p* ≤ 0.0018) in all patients (*n* = 19, Table 3). Moreover, there were significant positive strong to very strong Spearman rank correlation coefficients between mean MVD_tumor_/ MVA_tumor_ for both readers and mean *f*_tumor_/ BF_tumor_/ BV_tumor_ in the 10 patients in whom histological microvessel analysis was performed (ρ ≥ 0.661, *p* ≤ 0.0376, Table 3).

There were no significant Spearman rank correlations between mean D_tumor_/ D*_tumor_ for both readers and mean BF_tumor_/ BV_tumor_ in all patients (│ρ│ ≤ 0.392, *p* ≥ 0.0972) or between MVD_tumor_/ MVA_tumor_ and mean D_tumor_/ D*_tumor_/ PEM_tumor_ in the 10 patients in whom histological microvessel analysis was performed (│ρ│ ≤ 0.527, *p* ≥ 0.1173). Also, the product *f*_tumor_ x D*_tumor_, which supposedly reflects capillary blood flow [32], was not significantly correlated to BF_tumor_/ BV_tumor_/ MVD_tumor_/ MVA_tumor_ (│ρ│ ≤ 0.200, *p* ≥ 0.5617).

Correlation coefficients between mean MVD_tumor_/ MVA_tumor_ for both readers and mean *f* were not significantly different from correlation coefficients between mean MVD_tumor_/ MVA_tumor_ and mean BF_tumor_ (*p* ≥ 0.4001, |Z-score| ≤ 0.840). Also, correlations between mean MVD_tumor_/ MVA_tumor_ and mean BF for both readers were not significantly different from correlations between mean MVD_tumor_/ MVA_tumor_ and mean BV_tumor_ (*p* ≥ 0.5652, |Z-score| ≤ 0.575).

D_tumor_, D*_tumor_, *f*_tumor_, BF_tumor_, and BV_tumor_ values did not differ significantly between moderately differentiated (G2) and poorly differentiated tumors (G3, *p* ≥ 0.4495).

MVA and MVA values were higher in non-neoplastic parenchyma than in tumors for all 4 patients in whom histological microvessel parameters were analyzed both in tumors and non-neoplastic parenchyma (*p* = 0.1250, Supplementary Figure 3).

**Fig. 1:**
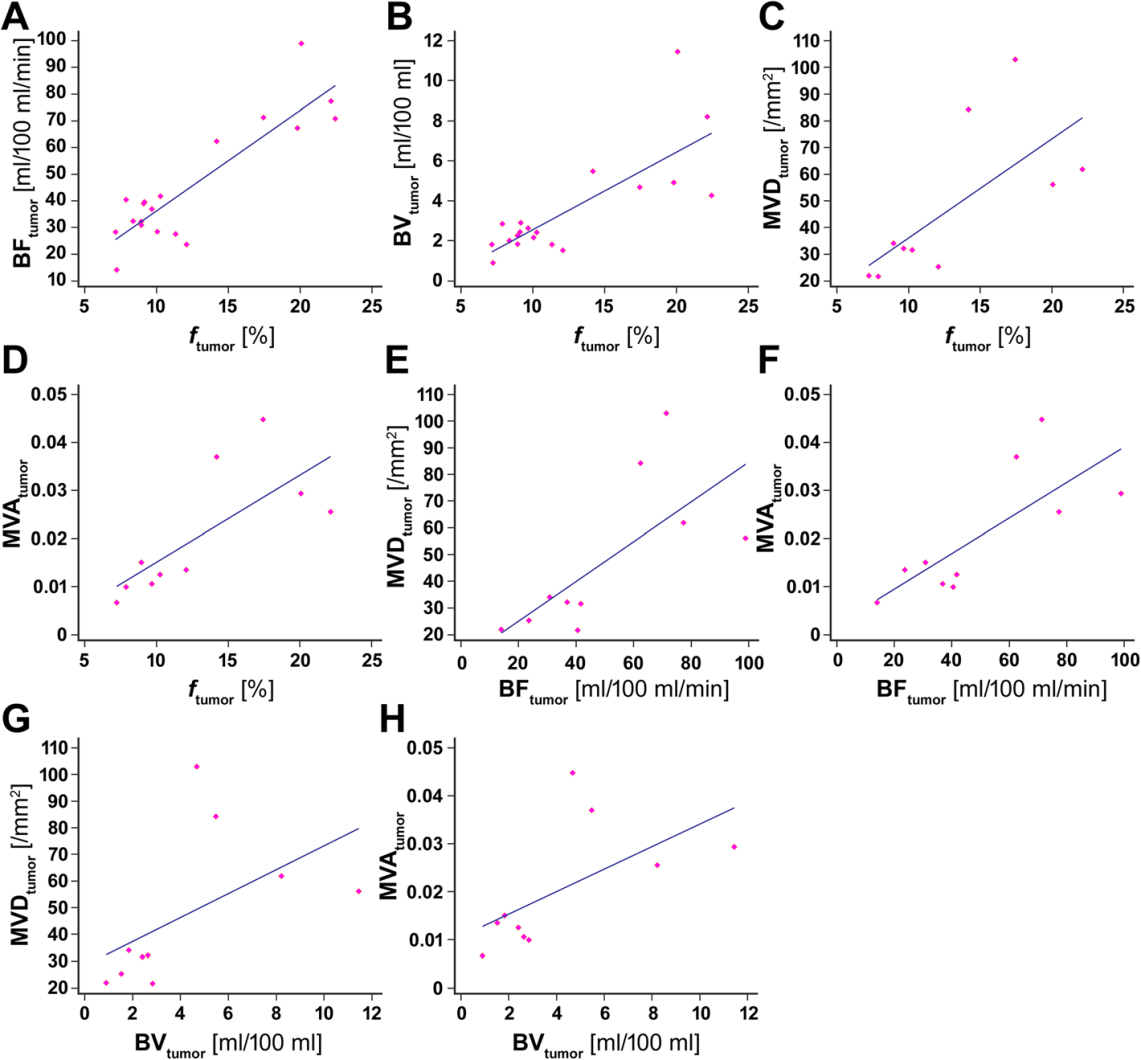
**Scatter plots with the results of the regression** **analysis. A**) Scatter plot of the perfusion fraction *f*_tumor_ vs. BF_tumor_. Linear regression model: ${BF}_{tumor}\left[\frac{ml}{100 ml\ast \mathit{\min}}\right]=3.772\frac{ml}{100 ml\ast \mathit{\min}\ast \%}\ast {f}_{tumor}\left[\%\right]-1.471\frac{ml}{100 ml\ast \mathit{\min}}$. Goodness of fit: *R*^2^ = 0.774. **B)** Scatter plot of the perfusion fraction *f*_tumor_ vs. BV_tumor_. Linear regression model: ${BV}_{tumor}\left[\frac{ml}{100 ml}\right]=0.388\frac{ml}{100 ml\ast \%}\ast {f}_{tumor}\left[\%\right]-1.327\frac{ml}{100 ml}$. Goodness of fit: *R*^2^ = 0.608**. C**) Scatter plot of the perfusion fraction *f*_tumor_ vs. MVD_tumor_. Linear regression model: ${MVD}_{tumor}\left[\frac{1}{mm^2}\right]=3.721\frac{1}{mm^2\ast \%}\ast {f}_{tumor}\left[\%\right]-1.144\frac{1}{mm^2}$. Goodness of fit: *R*^2^ = 0.476. D) Scatter plot of the perfusion fraction *f*_tumor_ vs. MVA_tumor_. Linear regression model: ${MVA}_{tumor}=0.00181\frac{1}{\%}\ast {f}_{tumor}\left[\%\right]-0.00306$. Goodness of fit: *R*^2^ = 0.548. **E**) Scatter plot of BF_tumor_ vs. MVD_tumor_. Linear regression model: ${MVD}_{tumor}\left[\frac{1}{mm^2}\right]=0.745\frac{100 ml\ast \mathit{\min}}{mm^2\ast ml}\ast {BF}_{tumor}\left[\frac{ml}{100 ml\ast \mathit{\min}}\right]+10.125\frac{1}{mm^2}$. Goodness of fit: *R*^2^ = 0.490. **F**) Scatter plot of BF_tumor_ vs. MVA_tumor_. Linear regression model: ${MVA}_{tumor}=0.00037\frac{100 ml\ast \mathit{\min}}{ml}\ast {BF}_{tumor}\left[\frac{ml}{100 ml\ast \mathit{\min}}\right]+0.00209$. Goodness of fit: *R*^2^ = 0.578. G) Scatter plot of BV_tumor_ vs. MVD_tumor_. Linear regression model: ${MVD}_{tumor}\left[\frac{1}{mm^2}\right]=4.489\frac{100 ml}{mm^2\ast ml}\ast {BV}_{tumor}\left[\frac{ml}{100 ml}\right]+28.346\frac{1}{mm^2}$. Goodness of fit: *R*^2^ = 0.281. H) Scatter plot of BV_tumor_ vs. MVA_tumor_.. Linear regression model: ${MVA}_{tumor}=0.00235\frac{100 ml}{ml}\ast {BV}_{tumor}\left[\frac{ml}{100 ml}\right]+0.0106$. Goodness of fit: *R*^2^ = 0.372

Scatter plots with the results of the regression analyses are shown in Fig. 1. The correlations between the displayed parameters *f*_tumor_, BF_tumor_, BV_tumor_, MVD_tumor_, and MVA_tumor_ are well visible.

**Fig. 2:**
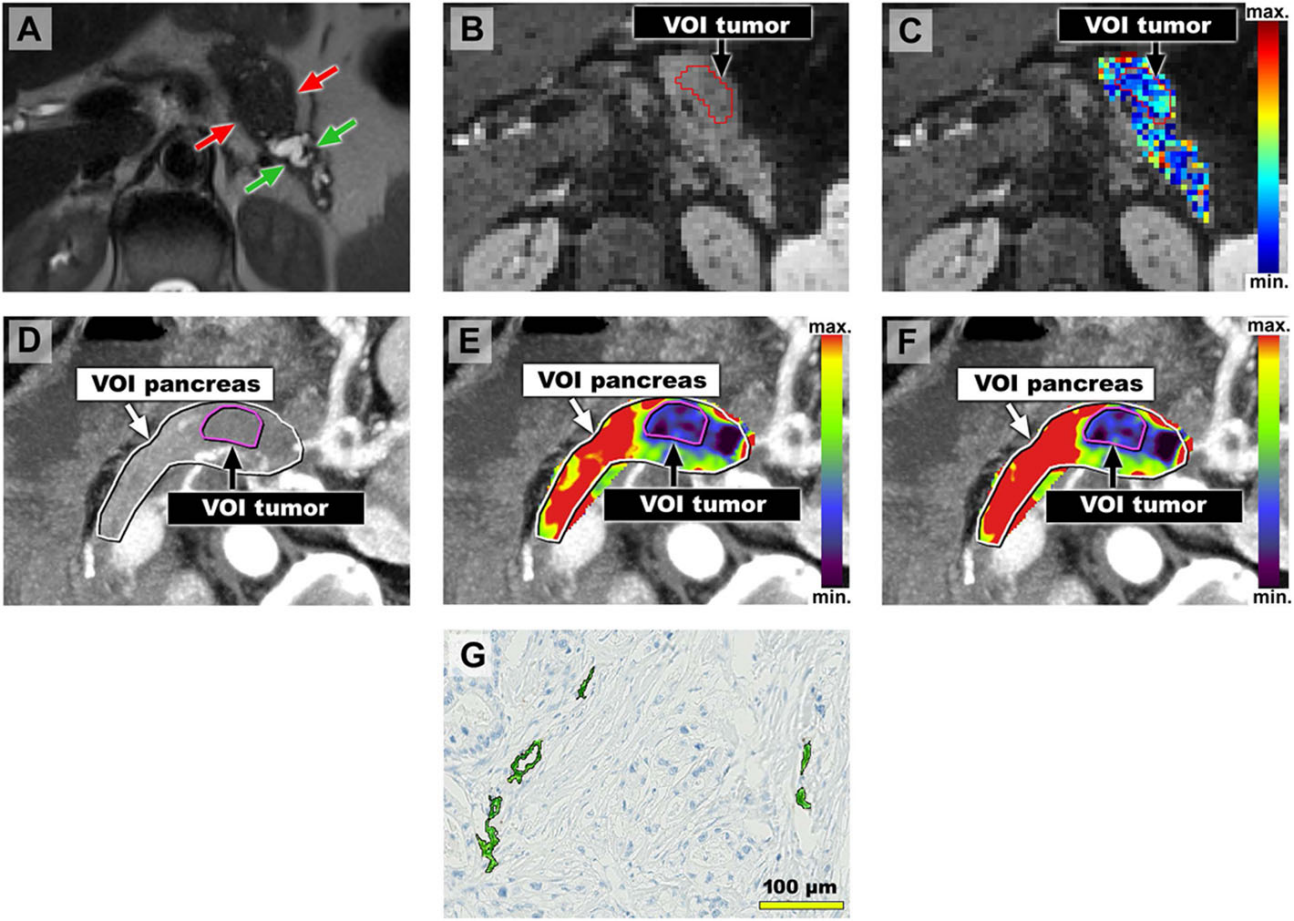
**Image examples of a patient with comparatively low tumor vascularity.** 64 years old female patient with PDAC of the pancreatic corpus/ tail. **a**-**c**) Axial MR images with the patient in supine position. **a** Axial T2-HASTE MR image shows upstream dilatation of the main pancreatic duct and concomitant parenchymal atrophy (green arrows) due to an obstructing tumor in the pancreatic corpus/ tail (red arrows). **b** Diffusion-weighted MR image (b = 300 s/mm^2^) with a VOI encompassing the tumor (VOI tumor). **c** Diffusion-weighted MR imaging with overlaying color-coded *f*-map. Mean calculated *f*_tumor_-value for both readers was low (8.9%). **d**–**f** Axial CT images with the patient in an oblique, 30°, right-sided down position. **d** Temporal maximum intensity projection (MIP) CT images of the perfusion sequence with VOIs encompassing the pancreas (VOI pancreas) and the tumor (VOI tumor). **e**-**f** Temporal MIP CT images with color-coded parameter maps for blood flow (BF_tumor_, **e**) and blood volume (BV_tumor_, **f**) derived from perfusion-sequence. Mean BF_tumor_ and BV_tumor_ were low (30.9 ml/100 ml/min and 1.8 ml/100 ml). **g**) Representative cutout of corresponding immunostained tissue slide (CD34) after semi-automated segmentation of microvessels shows low MVD_tumor_. MVD_tumor_ in total analysis area was 34.1 /mm^2^

**Fig. 3:**
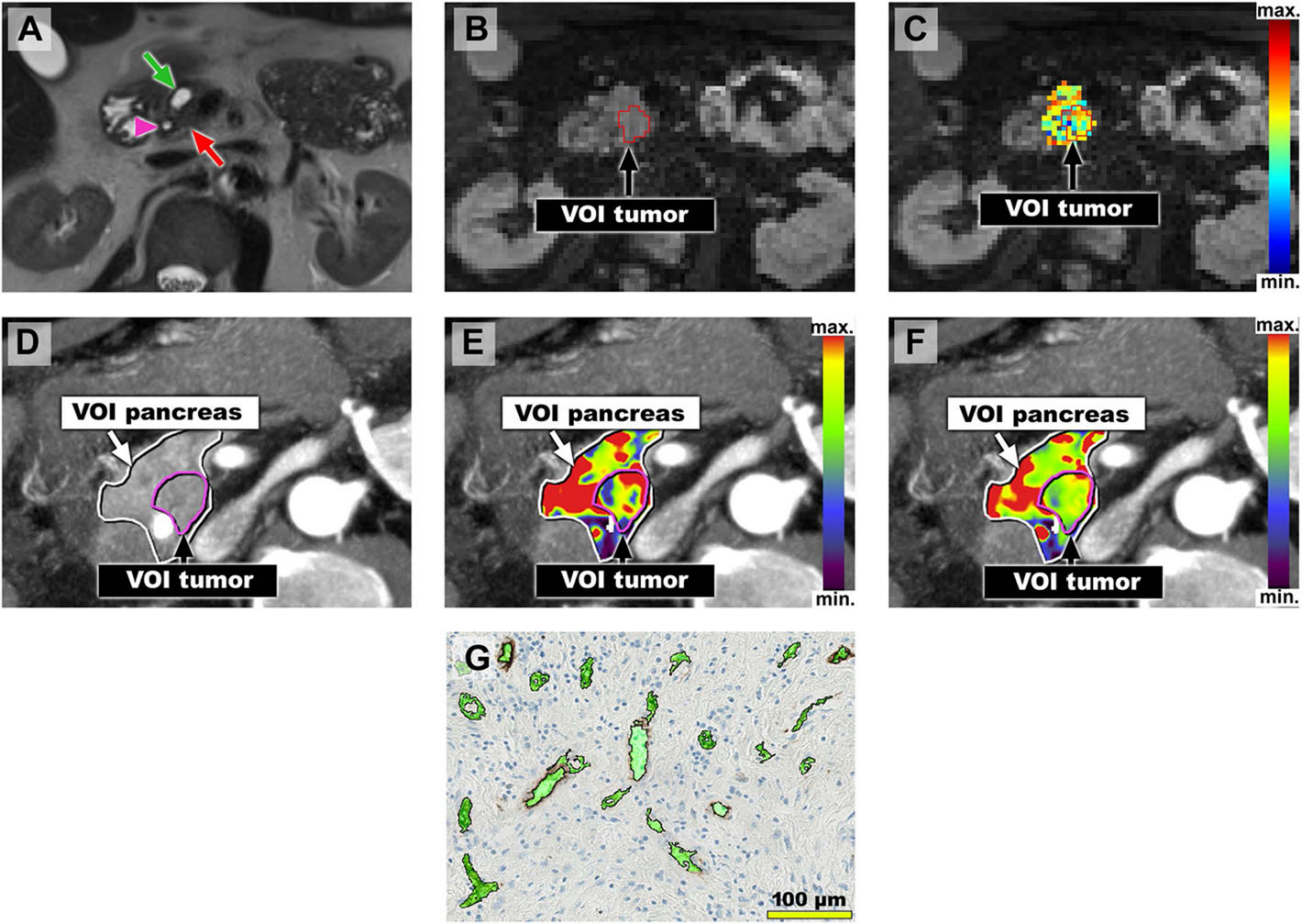
Image examples of a patient with comparatively high tumor vascularity. 58 years old male patient with PDAC of the pancreatic head. **a**-**c** Axial MR images with the patient in supine position. **a** Axial T2-HASTE MR image shows upstream dilatation of the main pancreatic duct (green arrow) due to an obstructing tumor in the pancreatic head (red arrow). Stent in the distal common bile duct which is not dilated (pink arrowhead). **b** Diffusion-weighted MR image (b = 300 s/mm^2^) caudad to the T2-HASTE image with a VOI encompassing the tumor (VOI tumor). **c** Diffusion-weighted MR imaging with overlaying color-coded *f*-map. Mean calculated *f*_tumor_-value for both readers was high (22.1%). **d**–**f** Axial CT images with the patient in an oblique, 30°, right-sided down position. **D**) Temporal maximum intensity projection (MIP) CT images of the perfusion sequence with VOIs encompassing the pancreas (VOI pancreas) and the tumor (VOI tumor). **e**–**f** Temporal MIP CT images with color-coded parameter maps for blood flow (BF_tumor_, **e**) and blood volume (BV_tumor_, **f**) derived from perfusion-sequence. Mean BF_tumor_ and BV_tumor_ were comparatively high (77.3 ml/100 ml/min and 8.2 ml/100 ml). **g** Representative cutout of corresponding immunostained tissue slide (CD34) after semi-automated segmentation of microvessels shows high MVD_tumor_. MVD_tumor_ in total analysis area was 61.8/mm^2^

Example pictures of PDAC patients with low and high perfusion/microvasculature are shown in Figs. 2 and 3.

## Discussion

The presented study evaluates tumor tissue perfusion as possible imaging biomarker in PDAC. In PDAC patients, tumor tissue perfusion was quantified by means of IVIM DW MRI as well as CT perfusion, and correlated with histologically determined MVD_tumor_/ MVA_tumor_.

We used the deconvolution model for CT perfusion analysis since it can tolerate greater image noise than compartment models and was reported to be well suited for measuring lower levels of perfusion [33], as expected in PDAC. The deconvolution-based BF_tumor_ values obtained in our current study were similar to previously reported deconvolution-based BF_tumor_ values in PDAC [34], but considerably higher than previously reported BF_tumor_ values based on the maximum-slope approach in PDAC [34, 35]. This difference between BF_tumor_ values obtained from the different models might be attributable to differences in the two mathematical calculation methods [34]. Mean BV_tumor_ and PEM_tumor_ values were within the same range as those reported by Schneeweiß et al. [34]. Also, mean values of the DWI IVIM parameters, as well as the histological microvessel parameters were within the same range as in a previous study by Klauß et al. [19].

The results of the current study confirm significant positive rank correlations between the IVIM-derived perfusion fraction *f*_tumor_ and MVD_tumor_/ MVA_tumor_ in PDAC. Similarly to a study by Klauß et al. there was no significant rank correlation between D*_tumor_ and MVD_tumor_ or MVA_tumor_, although D* is supposed to be a flow-related parameter [19]. A possible explanation for this observation could be linked to a study by Lemke et al. where the estimate of the pseudo-diffusion coefficient D* was found to be much less stable than the estimate of the perfusion fraction *f* [36]. As expected, the diffusion coefficient D_tumor_ did not significantly correlate with histological microvessel parameters in our present study. D represents perfusion-free diffusion [16] and was shown to reflect changes in the microarchitecture of the PDAC stroma [3].

The current study demonstrates that CT perfusion is a useful tool to evaluate tumor vascularity in PDAC. In the present study, BF_tumor_ and BV_tumor_ from CT perfusion similarly reflected MVD_tumor_ in PDAC. However, in a study on PNEN, BF_tumor_ significantly correlated with MVD_tumor_ whereas BV_tumor_ did not [18], which is in contrast to a study on colorectal cancer [37]. These discrepancies might be caused by differences in the microstructure of these tumor entities.

Rank correlations between microvessel parameters and PEM_tumor_ from CT perfusion were not significant in the current study. This is logical since PEM_tumor_ reflects features of microvessels like the microanatomy of the basement membrane rather than microvessel count [38]. Likewise, a study on PNEN failed to detect a significant correlation between PEM and MVD [18].

Probably the most important finding of the current study is that the applicability of DW IVIM MRI seems to be comparable to the applicability of CT perfusion for the assessment of tumor tissue perfusion. This finding is relevant for oncological imaging since tumor tissue perfusion can be used as imaging biomarker for treatment assessment [13]. This finding might also explain the excellent performance of both the perfusion fraction *f* from IVIM and CT perfusion parameters in distinguishing the notoriously hypovascular PDAC [39] from non-neoplastic pancreatic parenchyma in the present and previous studies [40, 41]. Valuable statements on the applicability of different radiological methods for the assessment of tumor vascularity can be accomplished only by direct comparison of methods and validation with histological microvessel parameters as ground truth. Similar studies should be performed in other solid tumors to further evaluate the interrelations of DW IVIM MRI, CT perfusion, and histological tumor microvascularity.

There are some limitations to our study. First, our patient sample is relatively small, although the time spent and effort per patient were comparatively high. Second, the complete series of tissue slides, to overcome the tumor heterogeneity, for histological and microvessel analysis were only available in 10 out of the 15 patients, who underwent surgery with curative intent for PDAC. Strikingly, this number of patients was still sufficient to prove significant correlations between microvessel parameters and *f*_tumor_ as well as BF_tumor_ and BV_tumor_. Third, the complete series of tissue slides was inspected in these 10 patients and representative tissue samples for microvessel analysis were chosen. However, these tissue samples weren’t completely congruent to the VOIs of the IVIM and CT perfusion analysis.

## Conclusion

In conclusion, this study shows that *f*_tumor_ and BF_tumor_/ BV_tumor_ similarly reflect microvasculature in PDAC and seem to be comparably applicable for the evaluation of tumor tissue perfusion for tumor characterization.

## Supplementary Information

**Additional file 1 Supplementary Figure 1. Line diagrams of DWI IVIM and CT perfusion parameters of tumors, downstream parenchyma, and upstream parenchyma.** Line diagrams depicting (A) *f*-values, (B) D-values, (C) D*-values, D) BF-values, E) BV-values, and F) PEM-values from Reader 1 (P.M.). Each line represents one patient.

**Additional file 2 Supplementary Figure 2. ROC curves for differentiation of tumors from upstream parenchyma.** ROC-curves for differentiation of tumors from upstream parenchyma using DWI IVIM and CT perfusion parameters from Reader 1. AUC-values were 0.874 (95% CI: 0.715 to 0.962) for *f*, 0.737 (95% CI: 0.558 to 0.872) for D, 0.514 (95% CI: 0.337 to 0.688) for D*, 0.937 (95% CI: 0.797 to 0.991) for BF, 0.902 (95% CI: 0.750 to 0.977) for BV and 0.888 (95% CI: 0.732 to 0.970) for PEM.

**Additional file 3 Supplementary Figure 3. Microvessel analysis in non-neoplastic parenchyma.** A) Representative cutout of immunostained non-neoplastic pancreatic tissue (CD34) after semi-automated segmentation of microvessels shows relatively high microvessel density. Segmentation of microvessels was performed using Aperio Microvessel Analysis software, with CD34 positive endothelial cells surrounding a (slit-like) lumen. Dotted CD34 expressions are mast cells, dendritic cells, and activated stroma cells. B) and C) Line diagrams depicting MVD and MVA values in non-neoplastic pancreatic parenchyma and tumors. Each line represents one patient. MVD_non-neoplastic_ and MVA_non-neoplastic_ values were higher than corresponding MVD_tumor_ and MVA_tumor_ values in all 4 patients (*p* = 0.1250).

**Additional file 4 Supplementary Table 1. Spearman rank correlation coefficients between all tumor DWI IVIM parameters, CT perfusion parameters, and histological microvessel parameters in tumors.**

## Data Availability

The datasets generated during and/or analysed during the current study are available from the corresponding author on reasonable request.

## Abbreviations

BF: Blood flow
BV: Blood volume
CI: Confidence interval
CT: Computed tomography
CTDI_vol_: Volumetric CT dose index
D: Diffusion coefficient
D*: Pseudodiffusion coefficient
D_eff_: Effective dose
DLP: Dose-length-product
DW: Diffusion weighted
DWI: Diffusion weighted imaging
*f*: perfusion fraction
IQR: Interquartile range
IVIM: Intravoxel Incoherent Motion
MITK: Medical Imaging Interaction Toolkit
MRI: Magnetic resonance imaging
MVA: Microvessel area
MVD: Microvessel density
PDAC: Pancreatic ductal adenocarcinoma
PEM: Permeability values
PNEN: Neuroendocrine neoplasm
SMA: Superior mesenteric artery
UICC: Union Internationale Contre le Cancer
VOI: Volume of interest
WHO: World Health Organization
