# Supplementary figures and images for: Assessment of tissue perfusion of pancreatic cancer as potential imaging biomarker by means of Intravoxel incoherent motion MRI and CT perfusion: correlation with histological microvessel density as ground truth
Source: Cancer Imaging. 2021 Jan 19;21:13. doi: 10.1186/s40644-021-00382-x (PMC7816417; doi:10.1186/s40644-021-00382-x)

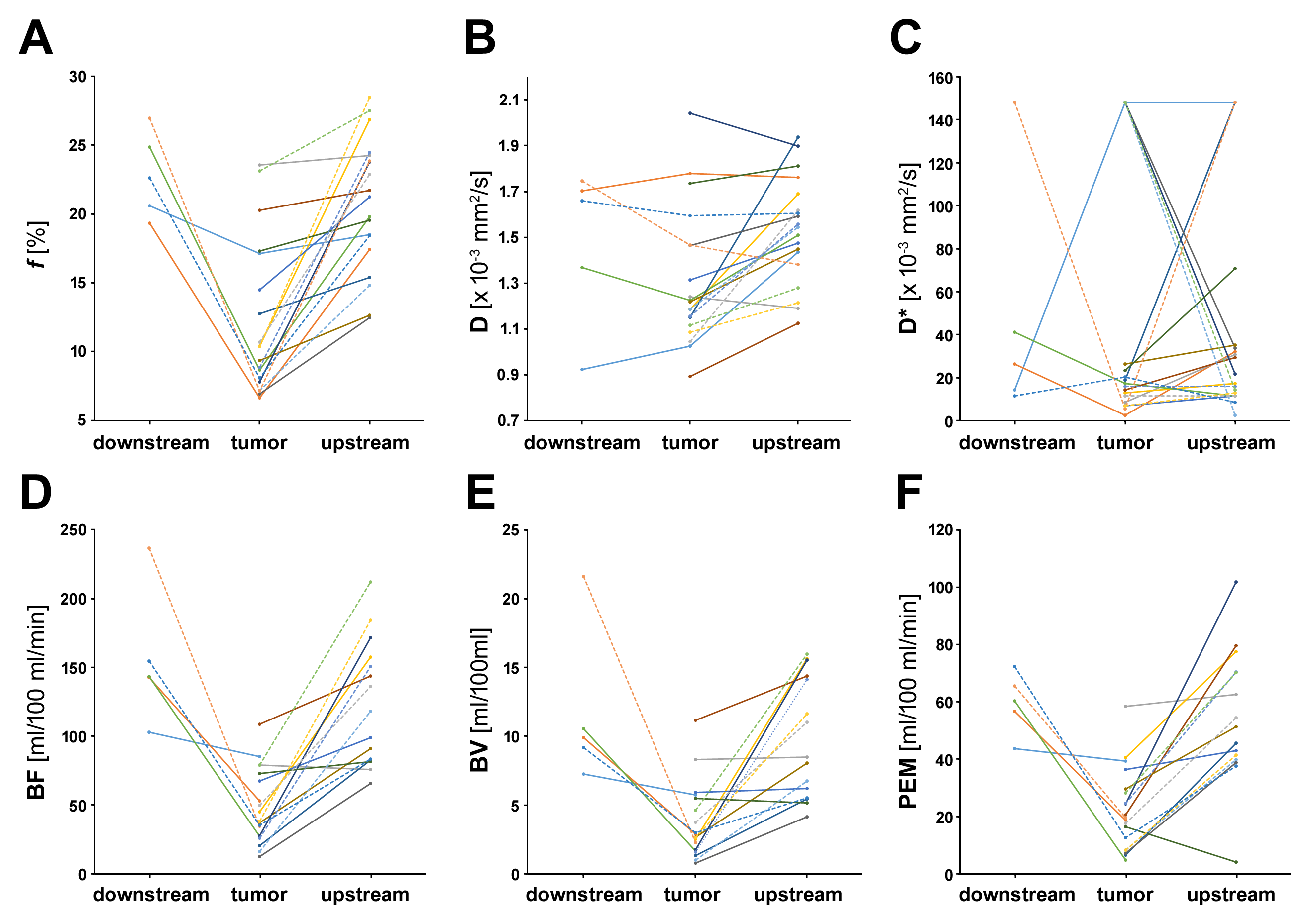

Supplement: Supplementary file 1 — Additional file 1 Supplementary Figure 1. Line diagrams of DWI IVIM and CT perfusion parameters of tumors, downstream parenchyma, and upstream parenchyma. Line diagrams depicting (A) f-values, (B) D-values, (C) D*-values, D) BF-values, E) BV-values, and F) PEM-values from Reader 1 (P.M.). Each line represents one patient. [file 40644_2021_382_MOESM1_ESM.tif]

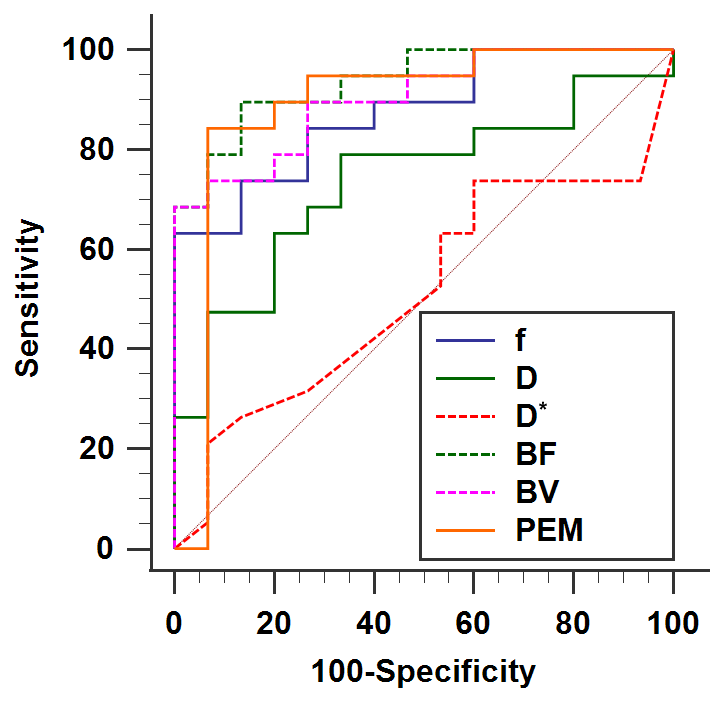

Supplement: Supplementary file 2 — Additional file 2 Supplementary Figure 2. ROC curves for differentiation of tumors from upstream parenchyma. ROC-curves for differentiation of tumors from upstream parenchyma using DWI IVIM and CT perfusion parameters from Reader 1. AUC-values were 0.874 (95% CI: 0.715 to 0.962) for f, 0.737 (95% CI: 0.558 to 0.872) for D, 0.514 (95% CI: 0.337 to 0.688) for D*, 0.937 (95% CI: 0.797 to 0.991) for BF, 0.902 (95% CI: 0.750 to 0.977) for BV and 0.888 (95% CI: 0.732 to 0.970) for PEM. [file 40644_2021_382_MOESM2_ESM.tif]

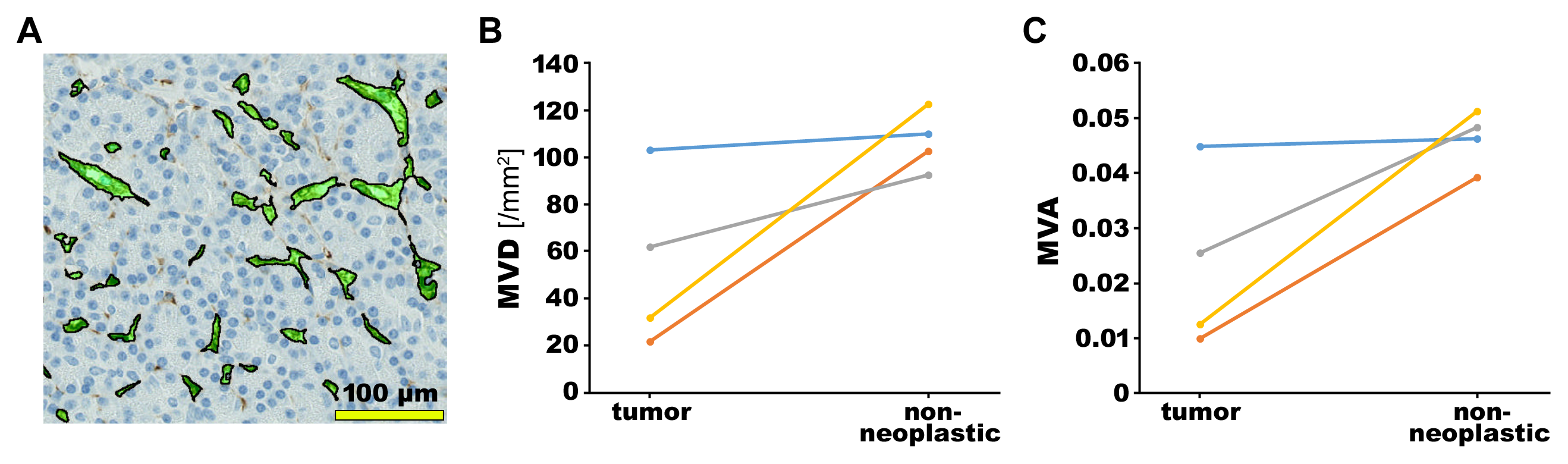

Supplement: Supplementary file 3 — Additional file 3 Supplementary Figure 3. Microvessel analysis in non-neoplastic parenchyma. A) Representative cutout of immunostained non-neoplastic pancreatic tissue (CD34) after semi-automated segmentation of microvessels shows relatively high microvessel density. Segmentation of microvessels was performed using Aperio Microvessel Analysis software, with CD34 positive endothelial cells surrounding a (slit-like) lumen. Dotted CD34 expressions are mast cells, dendritic cells, and activated stroma cells. B) and C) Line diagrams depicting MVD and MVA values in non-neoplastic pancreatic parenchyma and tumors. Each line represents one patient. MVDnon-neoplastic and MVAnon-neoplastic values were higher than corresponding MVDtumor and MVAtumor values in all 4 patients (p = 0.1250). [file 40644_2021_382_MOESM3_ESM.tif]
