# Supplementary material for: Assessment of tissue perfusion of pancreatic cancer as potential imaging biomarker by means of Intravoxel incoherent motion MRI and CT perfusion: correlation with histological microvessel density as ground truth
Source: Cancer Imaging. 2021 Jan 19;21:13. doi: 10.1186/s40644-021-00382-x (PMC7816417; doi:10.1186/s40644-021-00382-x)
Supplement: Supplementary file 4 — Additional file 4 Supplementary Table 1. Spearman rank correlation coefficients between all tumor DWI IVIM parameters, CT perfusion parameters, and histological microvessel parameters in tumors. [file 40644_2021_382_MOESM4_ESM.docx]

**Supplementary Table 1. Spearman rank correlation** **coefficients between all tumor DWI IVIM parameters, CT perfusion parameters, and histological microvessel parameters in tumors**

| **Spearman correlations between mean DWI IVIM parameters and mean CT perfusion parameters (n = 19)** | | | | | | | | |
| --- | --- | --- | --- | --- | --- | --- | --- | --- |
|  | BF_tumor_ | | | BV_tumor_ | | | PEM_tumor_ | |
| *f*_tumor_ | 0.668 * | | | 0.672 * | | | 0.398 | |
| D_tumor_ | -0.332 | | | -0.286 | | | -0.123 | |
| D*_tumor_ | -0.298 | | | -0.392 | | | -0.026 | |
| *f*_tumor_ x D*_tumor_ | -0.044 | | | -0.142 | | | 0.149 | |
| **Spearman correlations between histological microvessel parameters, mean DWI IVIM parameters and mean CT perfusion parameters (n = 10)** | | | | | | | | |
|  | *f*_tumor_ | D_tumor_ | D*_tumor_ | | *f*_tumor_ x D*_tumor_ | BF_tumor_ | BV_tumor_ | PEM_tumor_ |
| MVD_tumor_ | 0.770 * | -0.467 | -0.407 | | -0.200 | 0.697 * | 0.661 * | 0.527 |
| MVA_tumor_ | 0.818 * | -0.503 | -0.365 | | -0.139 | 0.709 * | 0.661 * | 0.382 |

Correlation coefficients that are significantly different from zero (p < 0.05) are marked with *. Abbreviations: BF: blood flow, BV: blood volume, D: diffusion coefficient, D*: pseudodiffusion coefficient, *f*: perfusion fraction, MVA: microvessel area, MVD: microvessel density, PEM: permeability.
